# Severe Outcomes Among Patients with Coronavirus Disease 2019 (COVID-19) — United States, February 12–March 16, 2020

**DOI:** 10.15585/mmwr.mm6912e2

**Published:** 2020-03-27

**Authors:** CDC COVID-19 Response Team, CDC COVID-19 Response Team, Stephanie Bialek, Ellen Boundy, Virginia Bowen, Nancy Chow, Amanda Cohn, Nicole Dowling, Sascha Ellington, Ryan Gierke, Aron Hall, Jessica MacNeil, Priti Patel, Georgina Peacock, Tamara Pilishvili, Hilda Razzaghi, Nia Reed, Matthew Ritchey, Erin Sauber-Schatz

**Affiliations:** CDC; CDC; CDC; CDC; CDC; CDC; CDC; CDC; CDC; CDC; CDC; CDC; CDC; CDC; CDC; CDC; CDC.

*On March 18, 2020, this report was posted online as an *MMWR *Early Release.*

Globally, approximately 170,000 confirmed cases of coronavirus disease 2019 (COVID-19) caused by the 2019 novel coronavirus (SARS-CoV-2) have been reported, including an estimated 7,000 deaths in approximately 150 countries (*1*). On March 11, 2020, the World Health Organization declared the COVID-19 outbreak a pandemic (*2*). Data from China have indicated that older adults, particularly those with serious underlying health conditions, are at higher risk for severe COVID-19–associated illness and death than are younger persons (*3*). Although the majority of reported COVID-19 cases in China were mild (81%), approximately 80% of deaths occurred among adults aged ≥60 years; only one (0.1%) death occurred in a person aged ≤19 years (*3*). In this report, COVID-19 cases in the United States that occurred during February 12–March 16, 2020 and severity of disease (hospitalization, admission to intensive care unit [ICU], and death) were analyzed by age group. As of March 16, a total of 4,226 COVID-19 cases in the United States had been reported to CDC, with multiple cases reported among older adults living in long-term care facilities (*4*). Overall, 31% of cases, 45% of hospitalizations, 53% of ICU admissions, and 80% of deaths associated with COVID-19 were among adults aged ≥65 years with the highest percentage of severe outcomes among persons aged ≥85 years. In contrast, no ICU admissions or deaths were reported among persons aged ≤19 years. Similar to reports from other countries, this finding suggests that the risk for serious disease and death from COVID-19 is higher in older age groups.

Data from cases reported from 49 states, the District of Columbia, and three U.S. territories (*5*) to CDC during February 12–March 16 were analyzed. Cases among persons repatriated to the United States from Wuhan, China and from Japan (including patients repatriated from cruise ships) were excluded. States and jurisdictions voluntarily reported data on laboratory-confirmed cases of COVID-19 using previously developed data collection forms (*6*). The cases described in this report include both COVID-19 cases confirmed by state or local public health laboratories as well as those with a positive test at the state or local public health laboratories and confirmation at CDC. No data on serious underlying health conditions were available. Data on these cases are preliminary and are missing for some key characteristics of interest, including hospitalization status (1,514), ICU admission (2,253), death (2,001), and age (386). Because of these missing data, the percentages of hospitalizations, ICU admissions, and deaths (case-fatality percentages) were estimated as a range. The lower bound of these percentages was estimated by using all cases within each age group as denominators. The corresponding upper bound of these percentages was estimated by using only cases with known information on each outcome as denominators.

As of March 16, a total of 4,226 COVID-19 cases had been reported in the United States, with reports increasing to 500 or more cases per day beginning March 14 (Figure 1). Among 2,449 patients with known age, 6% were aged ≥85, 25% were aged 65–84 years, 18% each were aged 55–64 years and 45–54 years, and 29% were aged 20–44 years (Figure 2). Only 5% of cases occurred in persons aged 0–19 years.

**FIGURE 1:**
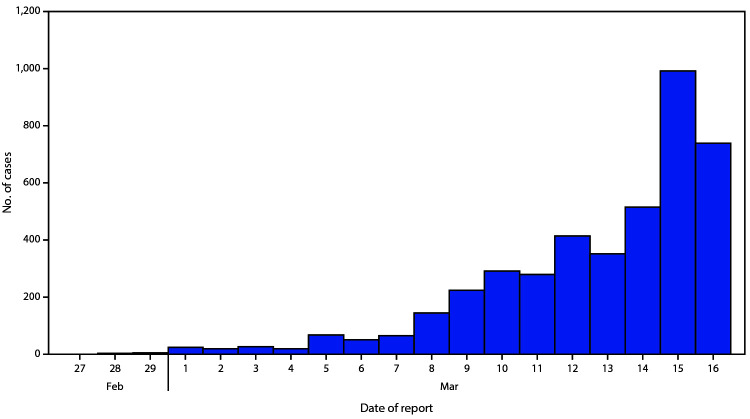
Number of new coronavirus disease 2019 (COVID-19) cases reported daily*^,†^ (N = 4,226) — United States, February 12–March 16, 2020 * Includes both COVID-19 cases confirmed by state or local public health laboratories, as well as those testing positive at the state or local public health laboratories and confirmed at CDC. ^†^ Cases identified before February 28 were aggregated and reported during March 1–3.

**Figure 2:**
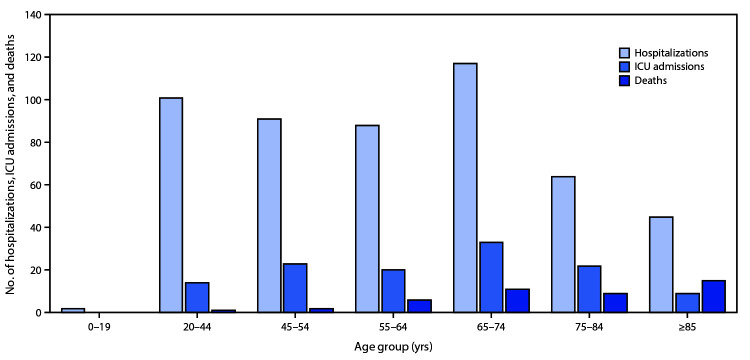
Coronavirus disease 2019 (COVID-19) hospitalizations,* intensive care unit (ICU) admissions,**^†^** and deaths,**^§^** by age group — United States, February 12– March 16, 2020 * Hospitalization status missing or unknown for 1,514 cases. ^†^ ICU status missing or unknown for 2,253 cases. ^§^ Illness outcome or death missing or unknown for 2,001 cases.

Among 508 (12%) patients known to have been hospitalized, 9% were aged ≥85 years, 36% were aged 65–84 years, 17% were aged 55–64 years, 18% were 45–54 years, and 20% were aged 20–44 years. Less than 1% of hospitalizations were among persons aged ≤19 years (Figure 2). The percentage of persons hospitalized increased with age, from 2%–3% among persons aged ≤19 years, to ≥31% among adults aged ≥85 years. (Table).

**TABLE:** Hospitalization, intensive care unit (ICU) admission, and case–fatality percentages for reported COVID–19 cases, by age group —United States, February 12–March 16, 2020

Age group (yrs) (no. of cases)	%*		
	Hospitalization	ICU admission	Case-fatality
0–19 (123)	1.6–2.5	0	0
20–44 (705)	14.3–20.8	2.0–4.2	0.1–0.2
45–54 (429)	21.2–28.3	5.4–10.4	0.5–0.8
55–64 (429)	20.5–30.1	4.7–11.2	1.4–2.6
65–74 (409)	28.6–43.5	8.1–18.8	2.7–4.9
75–84 (210)	30.5–58.7	10.5–31.0	4.3–10.5
≥85 (144)	31.3–70.3	6.3–29.0	10.4–27.3
**Total (2,449)**	**20.7–31.4**	**4.9–11.5**	**1.8–3.4**

* Lower bound of range = number of persons hospitalized, admitted to ICU, or who died among total in age group; upper bound of range = number of persons hospitalized, admitted to ICU, or who died among total in age group with known hospitalization status, ICU admission status, or death.

Among 121 patients known to have been admitted to an ICU, 7% of cases were reported among adults ≥85 years, 46% among adults aged 65–84 years, 36% among adults aged 45–64 years, and 12% among adults aged 20–44 years (Figure 2). No ICU admissions were reported among persons aged ≤19 years. Percentages of ICU admissions were lowest among adults aged 20–44 years (2%–4%) and highest among adults aged 75–84 years (11%–31%) (Table).

Among 44 cases with known outcome, 15 (34%) deaths were reported among adults aged ≥85 years, 20 (46%) among adults aged 65–84 years, and nine (20%) among adults aged 20–64 years. Case-fatality percentages increased with increasing age, from no deaths reported among persons aged ≤19 years to highest percentages (10%–27%) among adults aged ≥85 years (Table) (Figure 2).

## Discussion

Since February 12, 4,226 COVID-19 cases were reported in the United States; 31% of cases, 45% of hospitalizations, 53% of ICU admissions, and 80% of deaths occurred among adults aged ≥65 years with the highest percentage of severe outcomes among persons aged ≥85 years. These findings are similar to data from China, which indicated >80% of deaths occurred among persons aged ≥60 years (*3*). These preliminary data also demonstrate that severe illness leading to hospitalization, including ICU admission and death, can occur in adults of any age with COVID-19. In contrast, persons aged ≤19 years appear to have milder COVID-19 illness, with almost no hospitalizations or deaths reported to date in the United States in this age group. Given the spread of COVID-19 in many U.S. communities, CDC continues to update current recommendations and develop new resources and guidance, including for adults aged ≥65 years as well as those involved in their care (*7*,*8*).

Approximately 49 million U.S. persons are aged ≥65 years (*9*), and many of these adults, who are at risk for severe COVID-19–associated illness, might depend on services and support to maintain their health and independence. To prepare for potential COVID-19 illness among persons at high risk, family members and caregivers of older adults should know what medications they are taking and ensure that food and required medical supplies are available. Long-term care facilities should be particularly vigilant to prevent the introduction and spread of COVID-19 (*10*). In addition, clinicians who care for adults should be aware that COVID-19 can result in severe disease among persons of all ages. Persons with suspected or confirmed COVID-19 should monitor their symptoms and call their provider for guidance if symptoms worsen or seek emergency care for persistent severe symptoms. Additional guidance is available for health care providers on CDC’s website (https://www.cdc.gov/coronavirus/2019-nCoV/hcp/index.html).

This report describes the current epidemiology of COVID-19 in the United States, using preliminary data. The findings in this report are subject to at least five limitations. First, data were missing for key variables of interest. Data on age and outcomes, including hospitalization, ICU admission, and death, were missing for 9%–53% of cases, which likely resulted in an underestimation of these outcomes. Second, further time for follow-up is needed to ascertain outcomes among active cases. Third, the initial approach to testing was to identify patients among those with travel histories or persons with more severe disease, and these data might overestimate the prevalence of severe disease. Fourth, data on other risk factors, including serious underlying health conditions that could increase risk for complications and severe illness, were unavailable at the time of this analysis. Finally, limited testing to date underscores the importance of ongoing surveillance of COVID-19 cases. Additional investigation will increase the understanding about persons who are at risk for severe illness and death from COVID-19 and inform clinical guidance and community-based mitigation measures.*

The risk for serious disease and death in COVID-19 cases among persons in the United States increases with age. Social distancing is recommended for all ages to slow the spread of the virus, protect the health care system, and help protect vulnerable older adults. Further, older adults should maintain adequate supplies of nonperishable foods and at least a 30-day supply of necessary medications, take precautions to keep space between themselves and others, stay away from those who are sick, avoid crowds as much as possible, avoid cruise travel and nonessential air travel, and stay home as much as possible to further reduce the risk of being exposed (*7*). Persons of all ages and communities can take actions to help slow the spread of COVID-19 and protect older adults.^†^

**Summary**

**What is already known about this topic?**

Early data from China suggest that a majority of coronavirus disease 2019 (COVID-19) deaths have occurred among adults aged ≥60 years and among persons with serious underlying health conditions.

**What is added by this report?**

This first preliminary description of outcomes among patients with COVID-19 in the United States indicates that fatality was highest in persons aged ≥85, ranging from 10% to 27%, followed by 3% to 11% among persons aged 65–84 years, 1% to 3% among persons aged 55-64 years, <1% among persons aged 20–54 years, and no fatalities among persons aged ≤19 years.

**What are the implications for public health practice?**

COVID-19 can result in severe disease, including hospitalization, admission to an intensive care unit, and death, especially among older adults. Everyone can take actions, such as social distancing, to help slow the spread of COVID-19 and protect older adults from severe illness.

## Footnotes

* https://www.cdc.gov/coronavirus/2019-ncov/downloads/community-mitigation-strategy.pdf.

† https://www.whitehouse.gov/wp-content/uploads/2020/03/03.16.20_coronavirus-guidance_8.5x11_315PM.pdf.
